# Immunization with N2 neuraminidase can protect mice against a heterologous influenza A virus challenge even in the absence of cross-NA inhibiting antibodies

**DOI:** 10.1128/msphere.00300-26

**Published:** 2026-06-15

**Authors:** Laura Amelinck, Anouk Smet, Tine Ysenbaert, Koen Sedeyn, William Warren, Raul Gomila, Thorsten U. Vogel, Xavier Saelens, João Paulo Portela Catani

**Affiliations:** 1 Center for Medical Biotechnology, VIB, Ghent, Belgium; 2 Department of Biochemistry and Microbiology, Ghent University 26656, Ghent, Belgium; 3 Research North America, Sanofi, Waltham, Massachusetts, USA; University of Missouri, Columbia, Missouri, USA

**Keywords:** influenza vaccines, neuraminidase, N2

## Abstract

Neuraminidase (NA) inhibition (NAI) titers have been identified as an independent correlate of protection against influenza. Few studies, however, have investigated the breadth of NA-based immune protection. Previously, we have reported that N2 NAs derived from human H3N2 viruses that circulated between 2009 and 2017 can be subdivided into four antigenic groups. Here, we (i) immunized mice with recombinant soluble tetrameric NA from H3N2 strains representing those four antigenic groups or (ii) passively transferred N2 NA immune serum into naïve mice to evaluate the breadth of protection against a heterologous HxN2 influenza virus challenge. We show that the breadth of protection goes beyond the breadth of NAI and is associated with the presence of cross-reactive antibodies. Interestingly, in the absence of cross-reactive antibodies, the immunization of DBA/2J mice with heterologous recombinant N2 NA from A/Perth/16/2019 was associated with an early onset of disease upon challenge with HxN2 virus with NA from A/Indiana/08/2011 (H3N2) or with H2N2 A/Singapore/1/1957. This early onset of disease was unidirectional and was not observed when mice that had been immunized with recombinant NA from A/Indiana/08/2011 were challenged with HxN2 virus with NA from A/Perth/16/2009 (H3N2).

**IMPORTANCE:**

Although seasonal influenza vaccines mainly target hemagglutinin (HA), the viral surface protein neuraminidase (NA) can also induce protective immunity. In an earlier work, we profiled neuraminidase inhibition across a broad panel of H3N2 viruses and defined four major NA antigenic groups. Here, we tested whether immunizing mice with NA elicits cross protection against challenge viruses carrying NAs from different antigenic groups. Our data separate the predictive value of NA-binding and NA-inhibiting antibody responses and show that protection can extend beyond measurable NA inhibition when cross-reactive antibodies are present. These findings support the inclusion of standardized NA antigens as vaccine components to enhance the breadth of protection offered by seasonal influenza vaccine.

## INTRODUCTION

Hemagglutinin (HA) and neuraminidase (NA) are the major surface antigens of influenza virions. These two glycoproteins exert a complementary role in the influenza virus replication cycle: while HA binds to sialic acids, an essential step for virion entry, NA cleaves off terminal sialic acid residues (1). This balanced activity of HA and NA enables influenza virions to penetrate the mucus barrier, rich in decoy receptors, lining the respiratory tract, thereby enabling access to sialic acid receptors on the apical side of respiratory epithelial cells. Additionally, NA activity is crucial for the release of newly formed viral progeny from the infected cell (2, 3). NA-inhibiting antibodies can prevent or attenuate influenza by impairing these steps in the viral life cycle.

Traditionally, the protective humoral immune response against influenza virus infection has been attributed to the ability of antibodies to neutralize the virus by blocking attachment to sialic acid receptors present on the surface of susceptible cells. The presence of such neutralizing antibodies is typically evaluated with a hemagglutination inhibition assay performed with sera from immunized or convalescent subjects, red blood cells, and influenza virions. Hemagglutination inhibition (HAI) is accomplished by anti-HA antibodies and constitutes the gold standard correlate of protection against flu. Despite HA being the major target of influenza vaccines, studies from the 1970s already pointed out the protective effect of NA as an immunogen (4, 5). More recent studies have confirmed those early observations and established that anti-NA antibodies are an independent correlate of protection against influenza (6–8). The protective effect of NA inhibition has also been demonstrated in post-exposure prophylaxis: oseltamivir treatment protected close contacts of influenza virus-infected individuals (9). Despite NA’s protective potential, its immunogenicity is suboptimal in conventional split inactivated influenza vaccines, and NA is absent in recombinant hemagglutinin vaccines (10, 11).

Monoclonal antibodies can inhibit NA activity by occluding the catalytic site. Such antibodies prevent NA-mediated cleavage of small substrates, such as 2′-(4-methylumbelliferyl)-α-D-N-acetylneuraminic acid (MUNANA, a fluorogenic NA substrate) or NA-STAR (1,2-dioxetane derivative, a chemiluminescent substrate). Antibodies that bind NA without occluding its catalytic site can also interfere with NA activity, particularly when the substrate is a complex sialic acid-containing glycoprotein, such as fetuin; these antibodies impede access of the NA catalytic site by steric hinderance (12). Finally, NA-specific antibodies that do not inhibit NA activity can still protect mice from challenge with influenza virus. This protection relies on Fc effector functions, which are also involved in protection mediated by antibodies with weak NAI activity (13–15).

Previously, we determined the antigenic diversity of NA from human H3N2 viruses circulating from 2009 to 2017 by assessing ferret and mouse immune sera against a panel of HxN2 reassortant viruses in a fetuin-based NAI assay (16). The resulting NAI patterns revealed four antigenic groups (AGs) among the selected human H3N2 NAs (16). Here, using representative strains from each AG, we evaluated in mice the breadth of protection conferred by active immunization with an AF03-adjuvanted recombinant NA (N2) or passive transfer of NA immune sera against influenza virus challenge. Cross-protection was found to correlate with the presence of cross-reactive antibodies determined by ELISA.

## RESULTS

### Pathogenicity of HxN2 strains in mice

**Fig 1:**
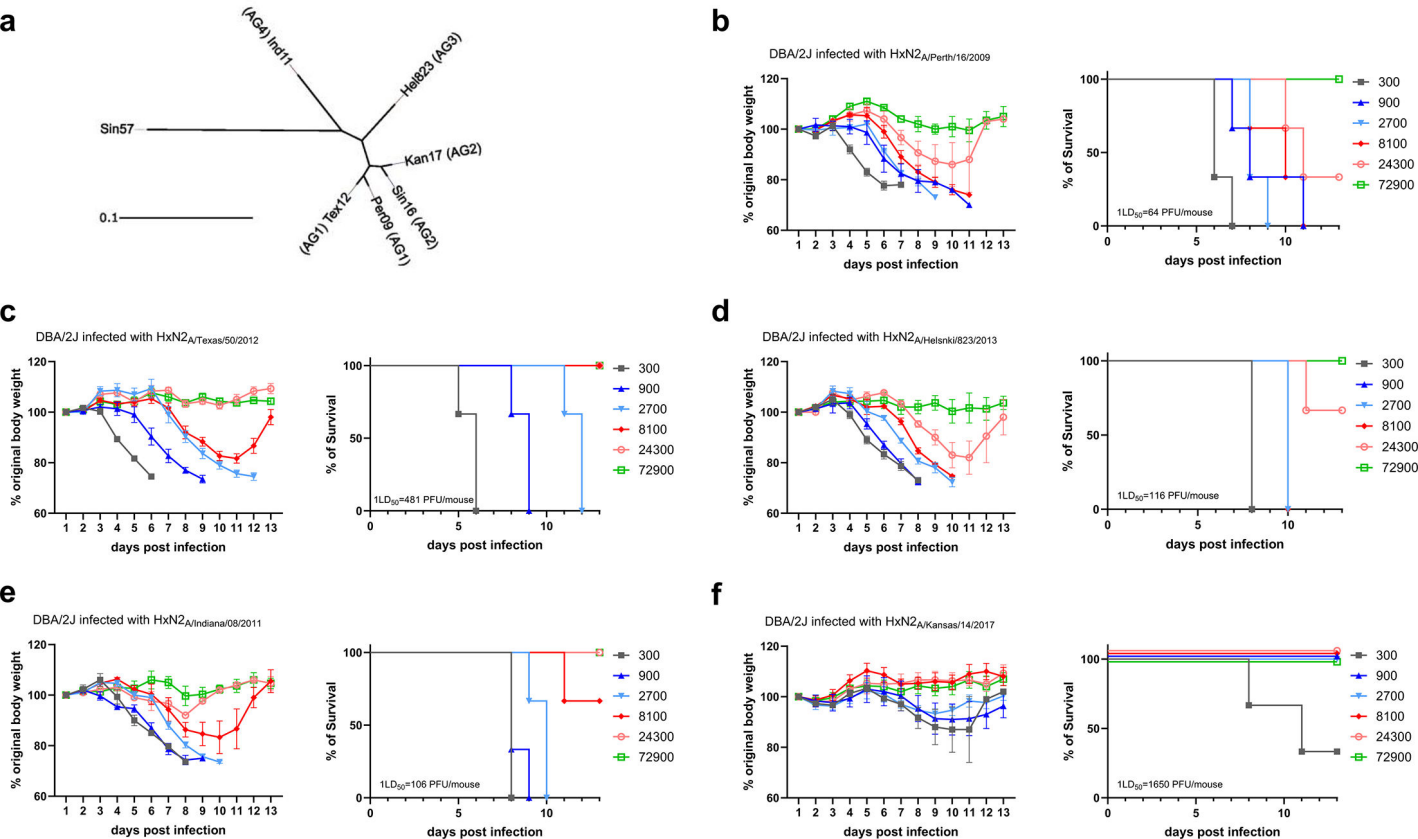
HxN2 reassortant viruses are pathogenic in DBA/2J mice. (**a**) Phylogenetic tree of NA amino acid sequence with color-coded AGs. Tex12 and Per09 in AG1; Sin16 and Kan17 in AG2; Hel823 in AG3, and Ind11 in AG4. The scale bar corresponds to 0.1 nucleotide substitutions per site. (**b–f**) Relative body weight, survival and LD_50_ of DBA/2J mice inoculated with (**b**) HxN2_A/Perth/16/2009nib-64_, (**c**) HxN2_A/Texas/50/2012_, (**d**) HxN2_A/Helsinki/823/2013_, (**e**) HxN2_A/Indiana/08/2011_, and (**f**) HxN2_A/Kansas/14/2017_. The LD_50_ values were calculated using the Reed–Muench method.

Five N2 NAs that represent the four AGs that we previously reported were selected for use as immunogens: NA from A/Perth/16/2009nib-64 (Per09) (AG1), A/Texas/50/2012 (Tex12) (AG1), A/Singapore/infimh-16-0019/2016 (Sin16) (AG2), A/Helsinki/823/2013 (Hel823) (AG3), and A/Indiana/08/2011 (Ind11) (AG4) (Fig. 1a) (16). Those N2 NA immunogens were produced as soluble tetrameric recombinant enzymatically active NA (tetNA) proteins in Chinese hamster ovary cells, have been described previously, and are referred to here as Per09 N2, Tex12 N2, Sin16 N2, Hel823 N2, and Ind11 N2 (16). To circumvent the poor pathogenicity of recent human H3N2 strains in mice, we established challenge models using H6N2 reassortant viruses, in which H6 (hereafter referred to as Hx) is derived from A/mallard/Sweden/81/2002 (H6N1). These six reassortant viruses are HxN2_Per09_, HxN2_Tex12_, HxN2_Sin16_, HxN2_Kan17_, HxN2_Hel823_, and HxN2_Ind11_ and have been described before (16). In an initial attempt to establish an HxN2 challenge model in mice, female BALB/c mice were inoculated with 10-fold serial dilutions of HxN2_Per09_. None of the mice, however, exhibited signs of disease or weight loss, even at the highest dose (8.2 × 10^3^ PFU per mouse) (Fig. S1a). DBA/2J mice are reported to have increased susceptibility to influenza virus infection (17). Therefore, the experiment was repeated in this mouse strain using threefold serial dilutions of HxN2_Per09_, HxN2_Tex12_, HxN2_Sin16_, HxN2_Kan17_, HxN2_Hel823_, and HxN2_Ind11_ (Fig. 1b through f). All HxN2 reassortant viruses were pathogenic in DBA/2J mice, except HxN2_Sing16_ (Fig. 1 and Fig. S1b).

### Protection induced by immunization with NA from distinct antigenic groups against HxN2_Per09_

Given that A/Perth/16/2009 (H3N2) has been used in a controlled human infection model, we first determined whether immunization with NAs from distinct AGs would protect mice against HxN2_Per09_ (18).

**Fig 2:**
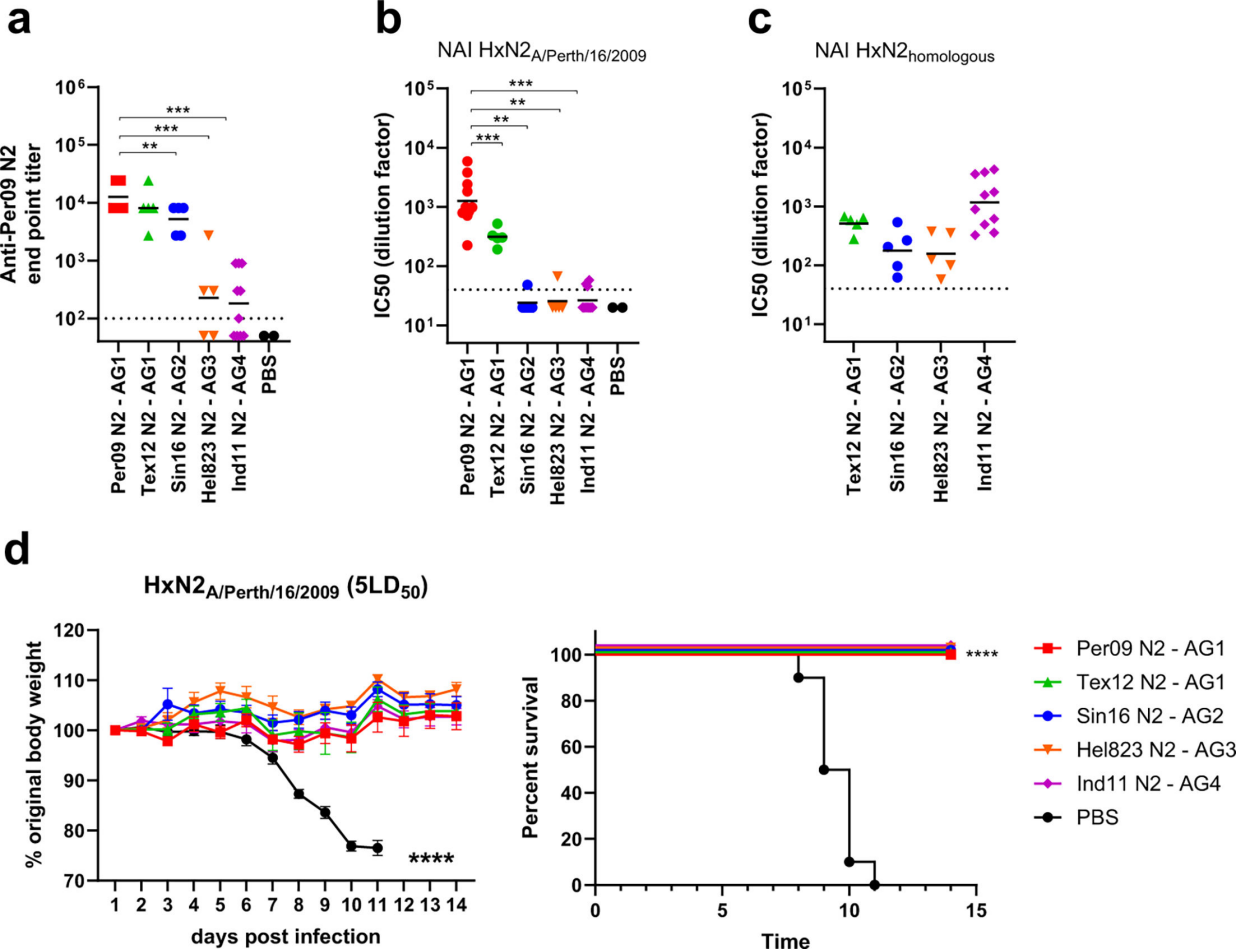
Serology and cross-protection of mice immunized with recombinant N2 and challenged with HxN_Pert09._ DBA/2J mice were prime-boosted in a 2-week interval with 0.1 µg of AF03-adjuvanted Perth09, Tex12, Sin16, Hel823, or Ind11 N2. Controls received AF03-PBS. (**a**) ELISA was performed with sera obtained 2 weeks after the boost using Perth09 N2 that was captured in nickel-coated plates. (**b and c**) NAI titers of the same sera against (**b**) HxN2_A/Perth/16/2009_ and (**c**) HxN2s with homologous NA (HxN2_Tex12_, HxN2_Sin16_, HxN2_Hel823_, and HxN2_Ind11_) used in the immunization. (**d**) Two weeks after the boost mice were challenged with 5LD_50_ of the reassortant HxN2_Per09_. The graphs show the percentage of initial body weight and mortality. ELISA endpoint titers were compared using Mann-Whitney test. NAI titers were compared using Welch’s *t*-test. Data points in panels **a–c** represent individual mice, except for the PBS group, which represent pooled sera from five mice per experiment. Horizontal solid lines represent the geometric means. Dashed lines represent the limit of detection (LOD). Values below the limit of detection were set to half the LOD (10 for NAI and 50 for endpoint titers). Data points in panel d represent means, and error bars indicate SEM. Relative body weights were analyzed as repeated measures using residual maximum likelihood (REML), and survival was analyzed with the Mantel-Cox (log-rank) test. Data in panel d are pooled from two independent experiments (*n* = 10), except for Tx12, NA Sin16, and Hel823 N2s, which are from one experiment (*n* = 5). **P < 0.01,***P < 0.001, and ****P < 0.0001 versus the PBS group.

Mice were primed and boosted with Per09 N2, Tex12 N2, Sin16 N2, Hel823 N2, and Ind11 N2, in a 2-week interval, and serum was collected, and viral challenge was performed 2 weeks after the boost. ELISA endpoint titers using microtiter plates coated with Per09 N2 (AG1) showed that homologous immunization induced the highest serum IgG titers (Fig. 2a). Tex12 N2, which belongs to AG1, also induced high titers against the coated Per09 N2 (Fig. 2a). Mice immunized with Sin16 N2 (AG2) had high titers of cross-reactive serum IgG, although these were significantly lower than those induced by homologous Per09 N2 immunization (Fig. 2a). Finally, mice immunized with Hel823 N2 (AG3) and Ind11 N2 (AG4) had significantly lower cross-reactive antibody titers against Per09 N2 (Fig. 2a). Low levels of antibodies directed against the tetrabrachion domain were detectable in the immune sera, which likely contributed to the observed cross-reactive serum IgG titers against the coated recombinant N2 proteins (Fig. S2) (19).

NAI titers of mouse immune sera were also measured against HxN2_Per09_. As expected, the highest NAI titers were observed in mice immunized with Per09 N2. NAI titers were also detected in the group immunized with the heterologous Tex12 N2, but these were significantly lower than those in the homologous group. Mice immunized with NA from different AGs—Sin16 N2, Hel13 N2, and Ind11 N2—had low or undetectable NAI titers against HxN2_Per09_ (Fig. 2b). To exclude the possibility that the reduced ELISA titers and NAI against Per09 reflected failed immunization, we also performed NAI assays using HxN2s homologous to each antigen. The homologous NAI titers confirmed that all groups responded as expected (Fig. 2c).

Two weeks after boost, mice were challenged with 5LD_50_ of HxN2_Per09_. All mice immunized with tetN2 were protected, and only the PBS control group exhibited weight loss and mortality (Fig. 2d).

### Protection induced by immunization with NA from A/Perth/16/2009 against HxN2 viruses with NA derived from distinct antigenic groups

Given that immunization with distinct NAs conferred protection against challenge with HxN2_Per09_, we next evaluated the reciprocal question: whether immunization with Per09 N2 could protect against challenge with HxN2 viruses bearing NA from distinct AGs. Four HxN2 viruses (HxN2_Tex12_-AG1, HxN2_Kan17_-AG2, HxN2_Hel823_-AG3, and HxN2_Ind11_-AG4) were used to challenge mice that had received homologous NA (matching the challenge virus NA; not available for HxN2_Kan17_, which received instead Sin16 N2, a NA from the same AG [AG2]), heterologous Per09 N2, or PBS.

**Fig 3:**
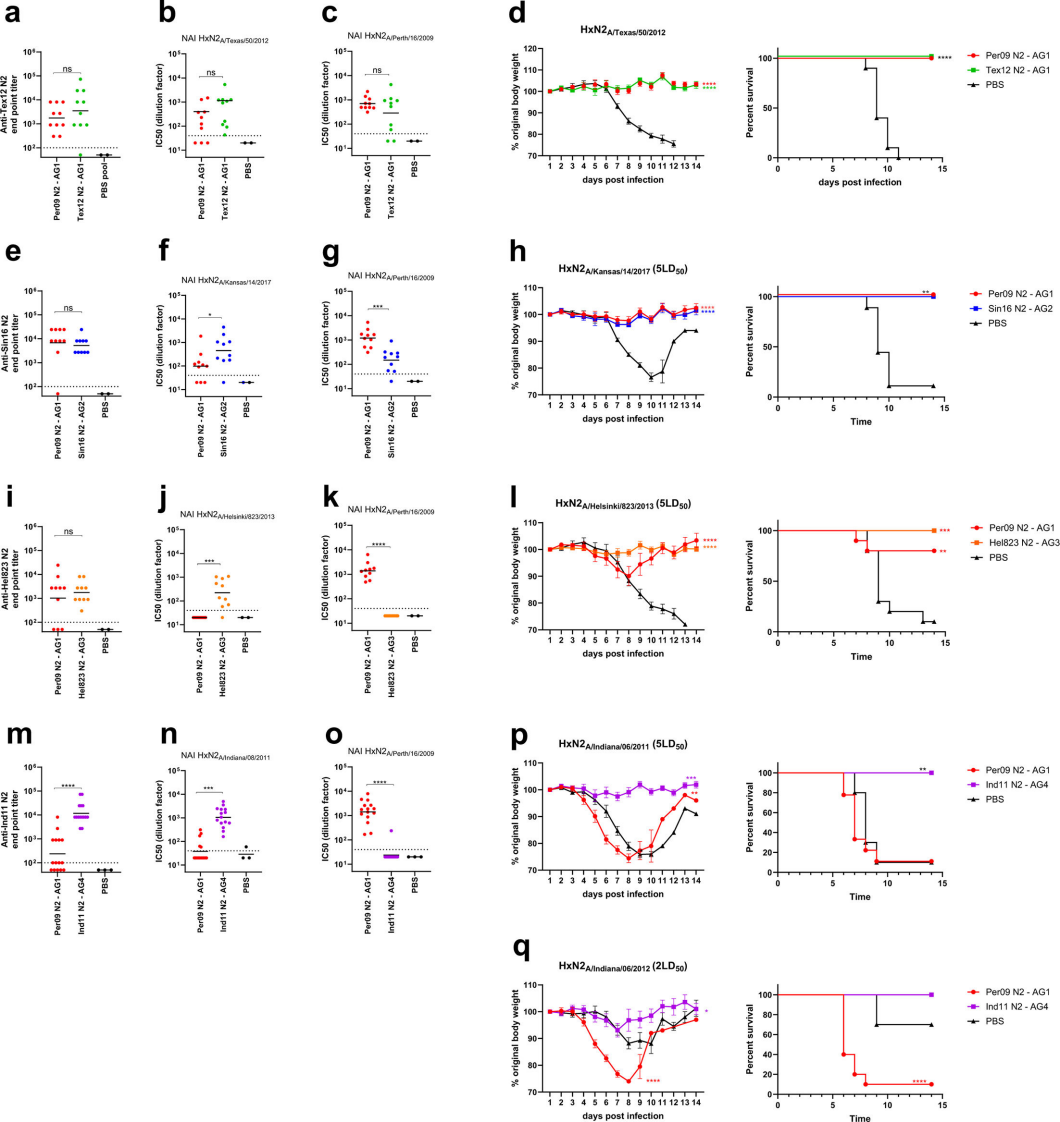
Serology and cross-protection of mice immunized with Per09 N2 and challenged with heterologous HxN2 reassortants. DBA/2J mice were prime-boosted in a 2-week interval with 0.1 µg of AF03-adjuvanted Perth09 N2, Tex12 N2, Sin16 N2, Hel823 N2, Ind11 N2, or AF03-PBS as indicated. ELISA and NAI were performed with sera isolated 2 weeks after the boost. (**a–d**) Per09 and Tex12 N2 comparison: (**a**) ELISA using Tex12 N2 on nickel-coated plates. (**b**) NAI against HxN2_Tex12_ and (**c**) HxN2_Per09_. (**d**) Relative body weight and survival following challenge with 5 LD_50_ of HxN2_Tex12_. (**e–h**) Per09 and Sin16 N2 comparison: (**e**) ELISA using Sin16 N2-coated plates. (**f**) NAI against HxN2_Kan17_ and (**g**) HxN2_Per09_. (**h**) Relative body weight and survival following challenge with 5LD_50_ of the HxN2_Kan17_. (**i-l**) Pet09 and Hel823 N2 comparison: (**i**) ELISA using Hel823 N2-coated plates. NAI determined against (**j**) HxN2_Hel823_ and (**k**) HxN2_Per09_. (**i**) Relative body weight and survival following challenge with 5LD_50_ of HxN2_Hel823_ virus. (**m–q**) Per09 and Ind11 N2 comparison: (**m**) ELISA using Ind11 N2. NAI determined against (**n**) HxN2_Ind11_ and (**o**) HxN2_Per09_. Relative body weight and survival of mice challenged with (**p**) 5 or (**q**) 2 LD_50_ of HxN2_Ind11_. ELISA endpoint titers were compared by Mann-Whitney test. NAI titers were compared using Welch’s t test. Horizontal solid lines represent the geometric means. Relative body weights were analyzed as repeated measures using residual maximum likelihood (REML), and survival was analyzed with the Mantel-Cox (log-rank) test. Dashed lines represent the limit of detection (LOD). Values below the limit of detection were set to half the LOD (10 for NAI and 50 for endpoint titers). All experiments were repeated once, and the data pooled for analysis (*n* = 10 mice per challenge). In panels **m–o**, 15 mice were included in the analysis. *P < 0.05, **P < 0.01, ***P < 0.001, and ****P < 0.0001 versus the PBS immunized group or as indicated.

The reciprocal experiment confirmed the antigenic similarity between Per09 and Tex12: immunization with Per09 N2 induced ELISA titers against Tex12 NA that were similar to titers induced by homologous Tex12 immunization (Fig. 3a). NAI titers of sera from Per09 N2-immunized mice against HxN2_Tex12_ were similar to those from Tex12 N2-immunized mice against HxN2_Per09_, confirming bidirectional cross-reactivity between these antigens (Fig. 3b and c). Consistent with these serologic data, both Per09- and Tex12 N2-immunized mice were protected against challenge with 5LD_50_ of HxN2_Tex12_ (Fig. 3d).

We next selected Sin16 NA because it belongs to AG2 and was a WHO-recommended vaccine strain in 2018-19 Northern Hemisphere (NH) season. Since HxN2_Sin16_ is not pathogenic in mice, HxN2_Kan17_ was used for challenge studies, as it belongs to the same AG2 as Sin16 N2. This AG2 is characterized by the substitutions S245N/S247T, and P468H in NA (16, 20). Anti-Sin16 N2 IgG titers measured 2 weeks after the boost were similar in mice immunized with Per09 N2 and Sin16 N2 (Fig. 3e). NAI titers against the HxN2_Kan17_ challenge strain were higher in mice immunized with Sin16 N2; however, detectable NAI titers were observed in sera from Per09 N2 immunized mice (Fig. 3f). Conversely, NAI titers against HxN2_Per09_ were higher in Per09 N2-immunized mice compared with Sin16 N2-immunized mice (Fig. 3g). Immunization with Per09 and Sin16 N2 equally protected mice against challenge with 5LD_50_ of HxN2_Kan17_ (Fig. 3h).

We also investigated protection induced by Per09 N2 against HxN2_Hel823_. Although isolated in 2013, this antigenically distinct AG3 N2 is genetically similar to NA from H3N2 viruses that circulated more than a decade earlier (16). Immunization with Per09 N2 induced cross-reactive IgG titers against Hel823 N2 that were comparable in magnitude to those obtained with homologous Hel823 N2 immunization (Fig. 3i). In contrast, sera from Per09 N2-immunized mice did not inhibit the NA activity of HxN2_Hel823_ (Fig. 3j). Similarly, sera from mice immunized with Hel823 N2 did not show detectable NAI against HxN2_Per09_ (Fig. 3k). Despite the lack of cross-reactive NAI titers, cross-protection after challenge was observed. Mice immunized with AG1 Per09 N2 showed transient weight loss and were similarly protected as the mice that had been immunized with homologous Hel823 N2 (Fig. 3i). This finding suggests that cross-reactive, non-inhibiting NA-specific antibodies can contribute to protection against heterologous NA challenge.

We next evaluated whether immunization with Per09 N2 (AG1) would confer protection against HxN2_Ind11_. Ind11 NA belongs to AG4 and is derived from a swine-origin H3N2 virus isolated from humans. Immunization with Per09 N2 induced low or undetectable cross-reactive ELISA titers against Ind11 N2, whereas all mice immunized with the homologous Ind11 N2 showed detectable titers (Fig. 3m). Similarly, NAI titers against HxN_Ind11_ were mostly absent or at lower levels in Per09 N2 immune sera compared with Ind11 N2-immunized mice (Fig. 3n). In line with this observation, NAI antibodies against HxN2_Per09_ were detected in Per09 N2-immunized mice but not in the Ind11 N2 immunized mice (Fig. 3o). Only mice that had been immunized with Ind11 N2 were protected against mortality and morbidity when challenged with 5 LD_50_ of HxN2_Ind11_. An early onset of morbidity was observed in the group immunized with heterologous Per09 N2 (Fig. 3p). To better evaluate this earlier onset of morbidity, we performed a similar experiment with a lower challenge dose (2LD_50_), which confirmed the observation (Fig. 3q).

### Protection mediated by N2 immune response is transferable by sera, even in the absence of NA inhibiting antibodies

Cross-protection against influenza can be mediated by cross-reactive antibodies or cellular responses directed against conserved antigens (2, 21–23). To evaluate whether protection observed after NA immunization relied on antibody responses, we performed serum transfer experiments in which naïve mice received immune sera from mice that had been immunized with NA representing AG1-4: Per09 N2, Sin16 N2, Hel823 N2, and Ind11 N2). Mice that had received the respective N2 immune sera were then challenged with 2LD_50_ of HxN2 viruses carrying NA from the respective AGs.

**Fig 4:**
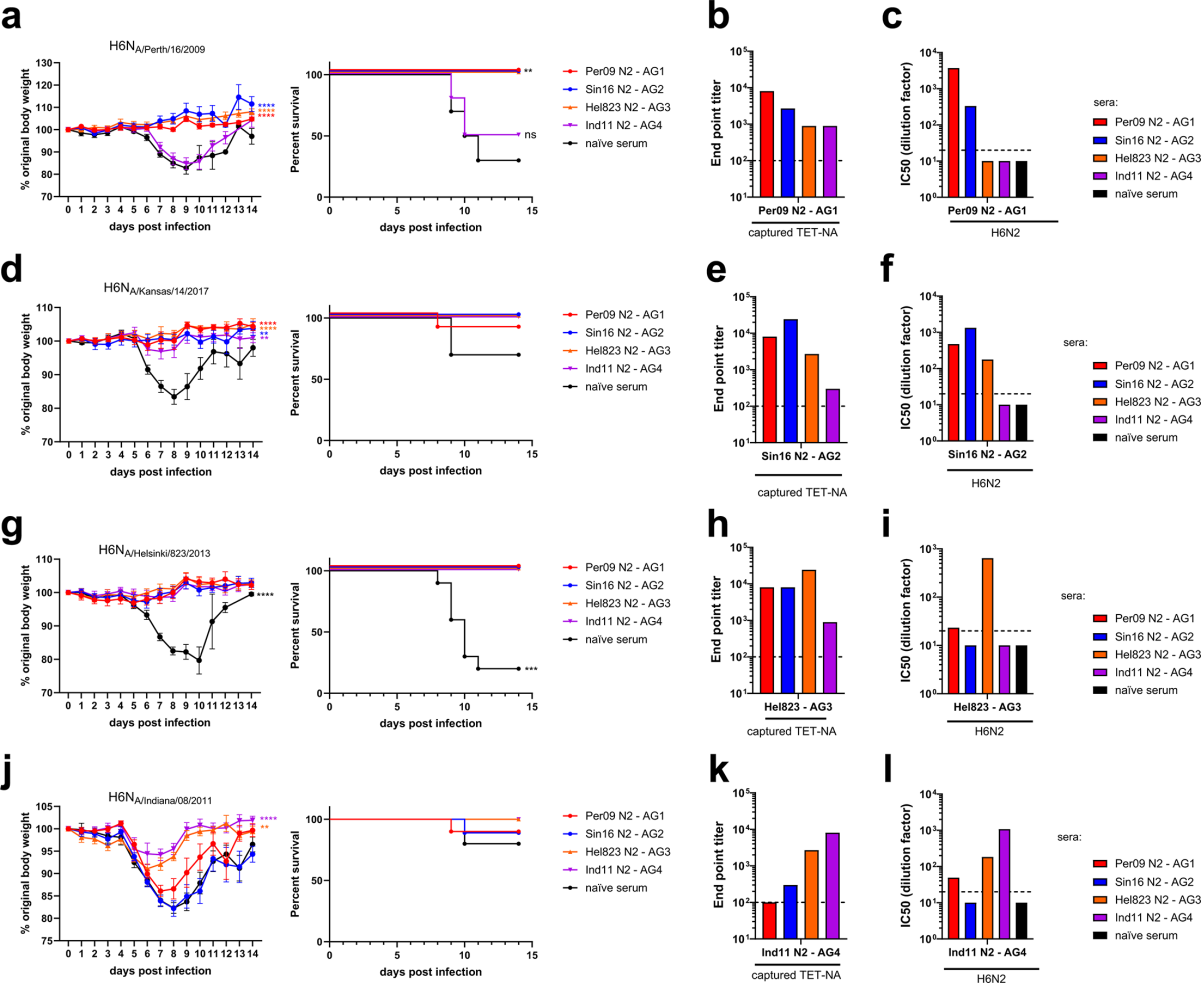
Passive protection by N2 NA immune serum against challenge with heterologous HxN2 viruses. Donor serum was obtained from DBA/2J mice immunized in a prime-boost regimen with 1 µg of AF03-Adjuvanted Per09 N2, Sin16 N2, Hel823 N2, Ind11 N2, or AF03-PBS (naïve serum). Recipient mice then received 100 µL of immune serum one day prior to challenge with the indicated HxN2 reassortant. (**a–c**) HxN2_Per09_ panel. (**a**) Relative body weight and survival after challenge with 2 LD_50_ of HxN2_Per09_. (**b**) ELISA using Per09 N2 captured in nickel-coated plates and (**c**) NAI titers against the HxN2_Per09_ challenge virus. (**d–f**) HxN2_Kan17_ panel. (**d**) Relative body weight and survival of mice challenged with 2 LD_50_ of HxN2_Kan17_. (**e**) ELISA using Sin16 N2 captured in nickel-coated plates and (**f**) NAI using HxN_Sin16_ virus, a reassortant with NA that belongs to the same AG as the NA of A/Kansas/14/2017. (**g–i**) HxN2_Hel823_ panel. (**g**) Relative body weight and survival of mice challenged with 2 LD_50_ of HxN2_Hel823_. (**h**) ELISA using Hel823 N2 captured in nickel-coated plates and (**i**) NAI against HxN2_Hel823_. (**j–l**) HxN2_Ind11_ panel. (**j**) Relative body weight and survival of mice challenged with 2 LD_50_ of HxN_Ind11_. (**k**) ELISA using Ind11 N2 captured in nickel-coated plates and (**l**) NAI titers against HxN2_Ind11_. Relative body weights were analyzed as repeated measures using residual maximum likelihood (REML), and survival was analyzed with the Mantel-Cox (log-rank) test. The passive serum transfer experiments followed by challenge were performed twice, and data from the two independent experiments were pooled for analysis (*n* = 10). Dashed lines represent the limit of detection. All ELISAs were performed on the pooled sera used for transfer. **P < 0.01; ****P < 0.0001 versus the naïve serum recipient group.

First, serum was generated by immunizing DBA/2J mice with 1 µg of N2 in a prime-boost regimen with a 2-week interval. Two weeks after the boost, immune serum was prepared, pooled per group, and analyzed by ELISA and NAI to confirm seroconversion prior to transfer to naïve mice. Mice that had received serum raised against Per09 N2, Sin16 N2, and Hel823 N2 were protected against challenge with HxN2_Per09_, whereas Ind11 N2 immune serum recipients were not (Fig. 4a). All transferred sera had detectable ELISA endpoint titers against Per09 N2 (Fig. 4b), whereas NAI titers against the HxN2_Per09_ challenge virus were detectable only in homologous Per09 N2 and heterologous Sin16 N2 immune sera (Fig. 4c). When challenged with 2LD_50_ of HxN2_Kan17_ (with AG2 NA), sera from the four AGs were protective against weight loss, while sera from naïve mice were not (Fig. 4d). Survival curves did not differ between any of the groups challenged with 2LD_50_ of HxN2_Kan17_ (Fig. 4d). All NA immune sera contained cross-reactive antibodies against Sin16 N2; however, only Ind11 N2 immune serum had undetectable NAI titers against HxN2_Sin16_ (Fig. 4e and f).

Mice that received any of the anti-N2 immune sera were protected against challenge with HxN2_Hel823_ (Fig. 4g). Although cross-reactive antibodies against Hel823 N2 were detected in all sera, only homologous sera had detectable NAI titers against HxN2_Hel823_ (Fig. 4h and i). Finally, most mice survived the challenge with 2LD_50_ of HxN2_Ind11_, and only mice that had received homologous Ind11 N2 or Hel823 N2 immune serum had reduced weight loss compared with naïve serum recipients (Fig. 4j). High titers of Ind11 N2 reactive antibodies were detected in Ind11 N2 and Hel823 N2 immune sera, whereas titers in Per09 and Sin16 N2 immune sera were near the limit of detection (Fig. 4k). Ind11 N2 and Hel823 N2 immune sera had high NAI titers against HxN2_Ind11_, whereas Per09 N2 and Sin16 N2 immune sera had low or undetectable NAI titers. The early onset of disease following challenge with HxN2Ind11 that was apparent in Per09 N2 immunized mice was not observed in the passive serum transfer experiment, suggesting that antibodies against a heterologous N2 NA are not sufficient or do not contribute to the enhanced disease in this mouse model (compare Fig. 3p and q with Fig. 4j).

### Immunization with Per09 N2 does not protect mice against A/Singapore/1/57 H2N2 challenge

There is evidence that the disease severity of the 1968 H3N2 pandemic influenza outbreak was mitigated by NA-specific immunity induced by prior exposure to H2N2 viruses in the population. We wanted to assess, in the DBA/2J mice, whether immunization with Per09 N2 could provide protections against challenge with A/Singapore/1/1957 (H2N2)(Sin57). Sin57 was not pathogenic in BALB/c mice and was to DBA/2J mice only at a very high inoculum (LD_50_ of 1.8 × 10^5^ PFU/mouse; Fig. S3). Therefore, we first performed mouse adaptation of Sin57. After 10 passages in mouse lungs and MDCK cells, the mouse-adapted virus exhibited increased pathogenicity, with 1LD_50_ of 4.6 × 10^2^ PFU/mouse. The mouse-adapted Sin57 carried a G103S and K422R substitution in HA and a G111D substitution in N2.

**Fig 5:**
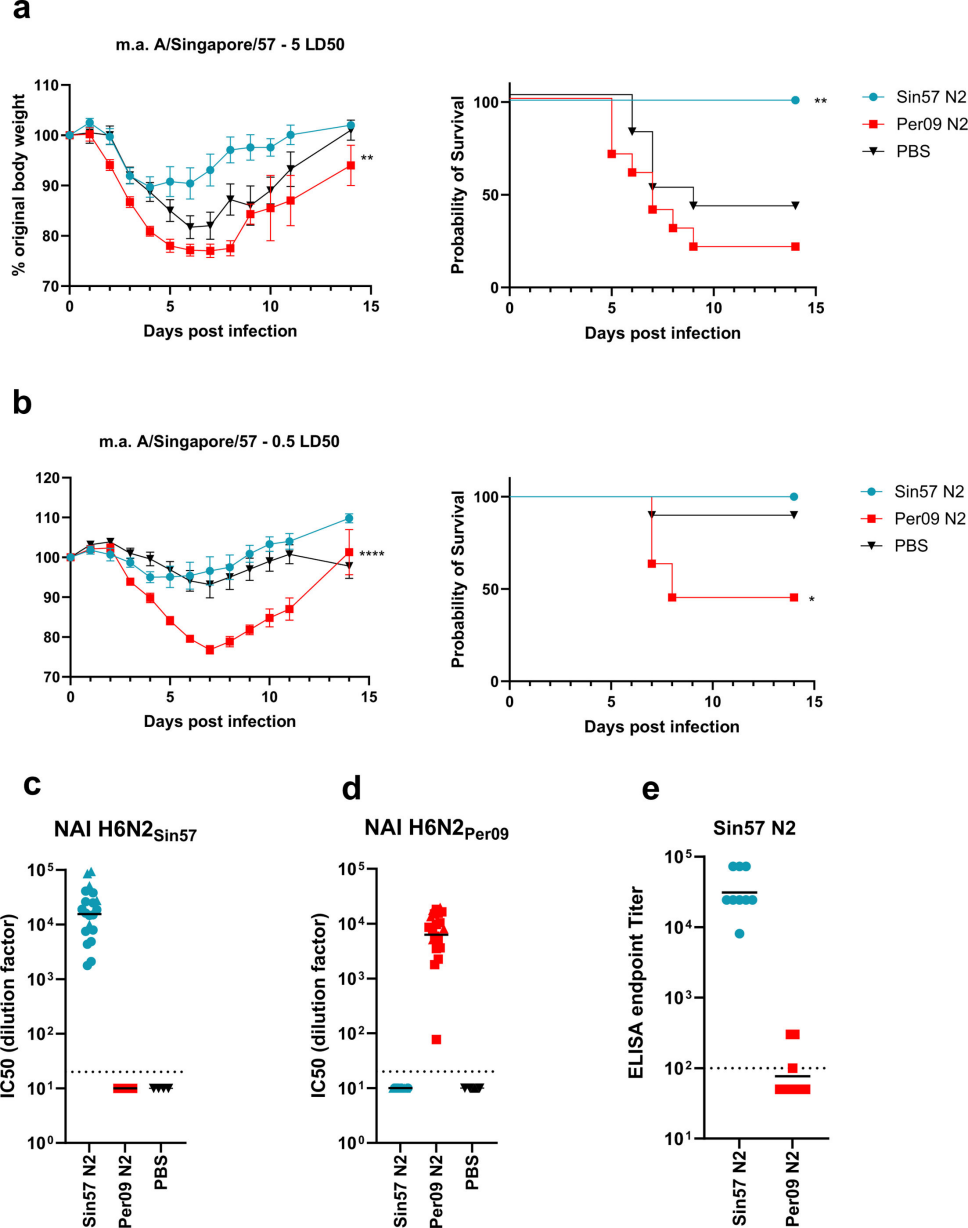
Immunization with Per09 N2 does not protect mice against A/Singapore/1/57 H2N2 challenge. DBA/2j mice were immunized in a prime-boost regimen with 0.1 µg of AF03-adjuvanted tetNA or AF03-PBS control. (**a and b**) Relative body weight and survival of mice after challenge with (**a**) 5LD_50_ or (**b**) 0.5LD_50_ of mouse-adapted Sin57. (**c and d**) NAI determined against (**c**) HxN2_Sin57_ or (**d**) HxN2_Per09_. (**e**) IgG titers determined by ELISA against Sin57 N2. Horizontal solid lines represent the geometric means. Dashed lines represent the limit of detection (LOD). Values below the limit of detection were set to half the LOD (10 for NAI and 50 for endpoint titers, respectively). Relative body-weight trajectories were analyzed as repeated measures using residual maximum likelihood (REML), and survival was analyzed with the Mantel-Cox (log-rank) test.. The experiments were performed twice, and data from the two independent experiments were pooled for analysis (*n* = 10). Dashed lines represent the limit of detection. *P < 0.05; **P < 0.01; ****P < 0.0001 versus the PBS immunized group.

Mice were immunized with homologous Sin57 N2 or heterologous Per09 N2. Two weeks after the boost, the mice were challenged with 5LD_50_ of mouse-adapted Sin57. Neither homologous nor heterologous NA immunization prevented infection, as all mice experienced at least transient weight loss following challenge. However, all mice immunized with homologous Sin57 N2 survived, whereas mice immunized with heterologous Per09 N2 were not protected (Fig. 5a). We also observed an early-onset morbidity and mortality trend in challenged mice immunized with Per09 N2. To further evaluate this observation, immunized mice were challenged with 0.5LD_50_ of mouse-adapted Sin57. While mice immunized with homologous Sin57 N2 or PBS control experienced a minor transient weight loss, the group immunized with heterologous Per09 NA displayed increased morbidity and mortality (Fig. 5b). No cross-NAI antibodies were detected against HxN2_Sin57_ and HxN2_Per09_ (Fig. 5c and d, respectively). In addition, most Per09 N2-immunized mice did not have detectable Sin57 N2 cross-reactive ELISA titers.

## DISCUSSION

Although NAI titers are an independent correlate of protection, NA has not yet been implemented as a standardized influenza vaccine component (24). This is due in part to the labile nature of NA and to our limited understanding of NA antigenicity relative to HA. Previously, we defined four distinct NA AGs in human H3N2 viruses isolated between 2009 and 2017 (16). Here, by active immunization of mice with AF03-adjuvanted N2 NAs representative of these four groups, we demonstrate that protection against an HxN2 virus challenge can occur in the absence of NA inhibiting antibodies.

To circumvent the poor pathogenicity of recent human H3N2 viruses in mice, we used HxN2 reassortants bearing H6 HA from A/mallard/Sweden/81/2002, N2 NAs from selected human H3N2 viruses, and the internal segments of the PR8 laboratory strain (25–27). Whereas inoculation of BALB/c mice with these HxN2 viruses did not induce signs of disease, DBA/2J mice proved susceptible to most HxN2 challenges, with LD_50_ values ranging from 64 to 1,650 PFU/mouse. Still, the HxN2_Sin16_ was not pathogenic, and HxN2_Kan17_ was pathogenic at doses 3.4 to 25 times higher than the other tested HxN2 viruses. Sin16 and Kan17 N2 belong to AG2, whose NAs exhibit a higher Km, potentially resulting in a suboptimal functional balance between H6 and N2 (16, 28).

After establishing challenge models with HxN2 representatives of the four NA AGs, we performed a series of active immunizations using AF03-adjuvanted NAs, followed by challenge. Our first question was whether antigenically distant N2 NAs would provide protection against HxN2_Per09_. Interestingly, despite the absence of detectable cross NAI titers, immunization with Sin16, Hel823, and Ind11 N2 fully protected mice against challenge with heterologous HxN2_Per09_. These immunizations induced cross-reactive antibodies against Per09 N2. This result was partially recapitulated by serum transfer experiments, in which all anti-N2 sera except Ind11 N2 protected against virus challenge. In line with this, active immunization with heterologous Per09 N2 also protected against heterologous HxN2_Tex12_, HxN2_Kan17_, and HxN2_Hel823_ challenge even in the absence of cross-NAI antibodies. Thus, the presence of cross-reactive antibodies sufficed to protect mice against heterologous HxN2 virus challenge, a finding recapitulated by serum transfer.

Intriguingly, mice immunized with Per09 N2 and challenged with heterologous HxN2_Ind11_ showed early-onset morbidity and mortality. This observation was not recapitulated by serum transfer experiments, in which only homologous Ind11 N2 and heterologous Hel823 N2 immune sera with detectable NAI were able to confer protection.

To assess whether the breadth of N2 NA-based immune protection could be expanded beyond the previously defined AGs, mice immunized with Per09 N2 or homologous N2 were also challenged with H2N2 Sin57. Similar to the HxN2_Ind11_ challenge experiments, immunization with heterologous Per09 N2 induced an early onset of morbidity and mortality after challenge with mouse-adapted Sin57, which was most evident at a sublethal dose. Per09 N2 immunization induced neither cross-inhibiting nor cross-reactive antibodies against H2N2 N2, consistent with increased divergency of Per09 N2 antigen and N2 from A/Singapore/1/1957 challenge (Fig. S4) and with the consequent lack of observed protection. Sequence comparison of the studied N2 relative to Per09 N2 plotted on the three-dimensional structure of NA highlights the impact of substitutions occurring near the catalytic pocket on NAI (Fig. S4) (16, 20). The increased divergence of Ind11 shows that a large proportion of epitopes (at least on the top face and lateral face of N2) are altered, which is consistent with the lower cross-reactivity observed in ELISA and may reflect antibodies targeting the tetrabrachion domain or bottom face of N2. The substantial divergency between Sin57 and Per09 largely agrees with the absence of cross-reactive antibodies.

The early onset of morbidity and mortality in the Per09 N2-immunized mice challenged with HxN2_ind11_ was not observed in the serum transfer experiments, suggesting a cellular mechanism may be at play. This is supported by the H2N2 challenge results, where a similar early onset of morbidity occurred even in the absence of cross-inhibiting and cross-reactive antibodies. Further research is required to elucidate the mechanisms underlying early disease in NA-immunized mice challenged with heterologous N2 NA-containing influenza viruses. Our data do not support this as a general phenomenon; rather, it is restricted to HxN2_ind11_ and H2N2 A/Singapore/01/1957 strains.

It is worth noting that some of the detected cross-reactive antibodies may be directed against the tetrabrachion domain. This zipper is immunogenic, yet not immunodominant, and may be less accessible in the capture ELISA used to determine NA-specific IgG titers (Fig. S2).

Overall, our data suggest that heterologous protection mediated by N2 NA-specific antibodies does not rely on cross-inhibiting antibodies but rather on cross-reactive antibodies.

While a number of clinical studies have identified NAI as an independent correlate of protection, with reported correlations to reduced viral shedding and shorter duration of symptoms (4, 6–8, 29–33), few studies have investigated whether this also applies to NA-binding antibodies. Notably, a recent study reported that NA-binding antibodies also correlate with protection from medically attended influenza caused by influenza A and B viruses (34). To our knowledge, no study has yet compared the predictive value of NAI and NA-binding titers. Based on our mouse data, we hypothesize that protection predicted from NA-binding titers may be broader than that predicted from NAI titers. These findings further encourage the use of NA as a standardized vaccine antigen to improve protection against influenza.

## MATERIALS AND METHODS

### Design and production of recombinant proteins

The coding information for tetNA fusion proteins (Per09, Tex12, Sin16, Hel823, Ind11, and Sin57 NAs) was cloned under the transcriptional control of the CMV promoter in the pCDNA3.4 plasmid with a CD5 secretion signal, an amino-terminal His-tag (except Sin57, which bears a strep-tag instead), and the thrombin cleavage signal, followed by tetrabrachion and NA head domain (truncated stalk at position 75). Per09, Tex12, Sin16, Hel823, and Ind11 NAs were produced in CHO cells, while Sin57 was produced in HEK293T cells. Recombinant proteins were purified by affinity followed by size exclusion chromatography as previously described (35).

### Viruses

The HxN2 reassortants were previously described in Catani et al. (16). Briefly, these reassortant viruses express the targeted NA antigen, internal genes from A/Puerto Rico/8/1934 H1N1 (PR8), and the HA from A/mallard/Sweden/81/2002 (H6N1). All segments were cloned into a bidirectional transcription plasmid derived from pUC57 (Genscript), including RNA polymerase (Pol) I and Pol II promoters, as previously described (36). The entire set of eight plasmids was used to transfect 293FT cells (Thermo Fisher Scientific) using Lipofectamine 2000 CD (Thermo Fisher Scientific). Twenty-four hours after transfection, Madin-Darby canine kidney cells (MDCK) ATL cells (ATCC) were added to the transfected cells in the presence of TPCK-treated trypsin (Sigma) to allow propagation of the rescued H6Nx viruses. Cell culture supernatants containing influenza virus were harvested 7 days post MDCK addition and blindly passaged in 8- to 10-day-old embryonated chicken eggs (Charles River Laboratories, Inc.). Inoculated eggs were incubated at 37°C for 48 h, then cooled to 4°C for 12 h, prior to allantoic fluid harvest and clarification by low-speed centrifugation (3,000 rpm, 20 min). High-yield stocks were generated by an additional passage in eggs as described above. Virus titers were determined by plaque assay on MDCK cells.

### ELISA

Anti-tetNA IgG titers were determined by capture ELISA using tetNAs in Pierce nickel-coated plates (cat. #15442). Recombinant NA proteins were diluted to 0.5 µg/mL in DPBS (Life Technologies, cat. #14040-182). Then, 50 µL of the coating antigen solutions was added to each well, and the plate was incubated at room temperature on a shaking platform (1 h for capture ELISA and overnight for conventional ELISA). The wells of the plates were then washed three times with PBS-T (Sigma, cat. #P3563-10PAK) and blocked for 1 h with 1% BSA in DPBS. After blocking, wells were washed once with PBS-T and incubated with a threefold serial dilution, starting from a 1/100 dilution, of serum in DPBS with 0.5% BSA and 0.05% Tween-20 for 2 h at room temperature on a shaking platform. Plates were then washed five times with PBS-T and incubated with a 1:5,000 dilution of anti-mouse IgG-HRP (GE Healthcare, cat. # NA931-1ml) in PBS with 0.5% BSA and 0.05% Tween-20. The 3,3′,5,5′-tetramethylbenzidine (TMB) substrate (BD, cat. #555214) was added after three washes with PBS-T, and the reaction was stopped after 5 min by addition of 50 µL of 1 M H_2_SO_4_. The optical density (OD) in each well was determined at 450 nm and, as a reference, 655 nm using an iMark Microplate Absorbance Reader (Bio-Rad). The end point titer was determined for each serum sample by scoring the dilution that resulted in an OD that was equal to or two times higher than the background OD obtained from pre-immune control sera dilution series.

### ELLA to determine NAI titers

Fetuin (Sigma cat. # F3385) was diluted into coating buffer (KPL cat. #50-84-01) to a concentration of 25 µg/mL, and 50 µL was added to the wells of Nunc MaxiSorp plates (Thermo Fisher cat. #44-2404-21), which were subsequently incubated overnight at 4°C. The coated plates were then washed three times with PBS-T (Sigma cat. #P3563-10PAK) and incubated overnight with 25 μL of a dilution of HxNx that corresponds to 70% of the maximum activity of NA from the respective viruses as determined in the ELLA, and 25 µL of two-fold serial dilution of serum, starting at a 1/20 dilution, in sample buffer (1× MES VWR cat. #AAJ61979-AP: 20 mM CaCl_2_, 1% BSA, 0.5% Tween-20). Fetuin-coated plates were then washed three times with PBS-T and incubated for 1 h with a solution of PNA-HRP (cat. #L6135-1MG, Sigma) at 5 μg/mL in conjugate diluent (MES pH 6.5, 20 mM CaCl_2_, 1% BSA). The plates were washed three times with PBS-T, TMB substrate was added, and then the plates were incubated for 5 min before the reaction was stopped by the addition of 50 µL of 1 M H_2_SO_4_. The optical density was measured at 450 nm and, as a reference, 655 nm in an iMark Microplate Absorbance Reader (Bio-Rad). Half maximum inhibitory concentration (IC_50_) values were determined by non-linear regression analysis using GraphPad Prism software.

### Animal experiments

Female DBA/2J mice, aged 7–8 weeks, were purchased from Janvier (France). The mice were housed in a specified pathogen-free animal house with food and water *ad libitum*. Animals were immunized intramuscularly in the right quadriceps. Immunization was performed with 50 µL containing the recombinant protein. Protein-based immunizations or PBS control was adjuvanted with a 1:1 volume of AF03 (25 μL of antigen in PBS + 25 µL of AF03 per dose) (37).

Blood samples were obtained by tail vein puncture two weeks after boost immunization. For virus inoculation, the mice were anesthetized with 5% isoflurane, and 50 μL of virus dilution was applied into both nostrils ensuring complete aspiration. Body weight was determined daily for 14 days after challenge. Mice were euthanized when they had lost more than 25% of body weight relative to the day of challenge.

For the serum transfer experiments, 100 μL of pooled sera prepared from mice that had been immunized with 1 μg of AF03-adjuvanted recombinant NA or PBS (with adjuvant) was injected intraperitoneally into DBA/2J mice 1 day prior to challenge.

H2N2 Sin57 was mouse-adapted by 10 passages through lung and MDCK cells. For each passage, groups of three DBA/2J mice were inoculated with a 1/100 dilution of the viral stock and sacrificed 72 h later to recover virus from the lungs. Lung homogenates were pooled and prepared in PBS using 7-mL tubes and a Precellys Evolution mechanical homogenizer, at 10,000 RPM for two cycles of 2 min. The homogenate was clarified by centrifugation at 1,000 × *g* for 10 min at 4°C, then diluted 1:1,000 prior to inoculation of MDCK cells. Infected cells were maintained in the presence of 2 µg/mL TPCK-treated trypsin (Sigma-Aldrich, T1426) until observation of cytopathic effect. The cell culture medium was then recovered, cleared by centrifugation, and used at 1/100 dilution to inoculate naive mice.

HxN2 challenge experiments were performed in BSL2, and experiments with A/Singapore/1/1957 were performed in a BSL3 laboratory.

### Statistical analysis

Relative body weight values were analyzed as repeated measurements using residual maximum likelihood (REML). Statistical analysis of survival was done using the log-rank (Mantel-Cox) test. ELISA titers were compared using the Mann-Whitney or Kruskal-Wallis test. NAI titers were compared using Welch’s t test or Kruskal-Wallis test. All statistical tests were performed using GraphPad Prism version 8.3.0. Statistical significance was considered when *P*  <  0.05.

## SUPPLEMENTAL MATERIAL

Supplemental Figures msphere.00300-26-s0001.docx Figures S1 to S4.

ASM does not own the copyrights to Supplemental Material that may be linked to, or accessed through, an article. The authors have granted ASM a non-exclusive, world-wide license to publish the Supplemental Material files. Please contact the corresponding author directly for reuse.
