# Supplementary material for: Immunization with N2 neuraminidase can protect mice against a heterologous influenza A virus challenge even in the absence of cross-NA inhibiting antibodies
Source: mSphere. 2026 Jun 15;11(7):e00300-26. doi: 10.1128/msphere.00300-26 (PMC13410959; doi:10.1128/msphere.00300-26)
Supplement: Supplemental Figures — Figures S1 to S4. [file msphere.00300-26-s0001.docx]

**Supplementary Figures**

**Supplementary Figure 1. Pathogenicity of HxN2 reassortant viruses in mice.** (**a**) Relative body weight of BALB/c mice inoculated with serial dilution of HxN2_Per09_. (**b**) Relative body weight of DBA/2J mice inoculated with a serial dilution of HxN2_Sin16_.

**Supplementary Figure 2. IgG titers against tetrabrachion zipper domain are induced by tetNA immunization with Per09 N2, Sin16 N2, Hel823 N2, and Ind11 N2.** Tetrabrachion domain fused to human serum albumin was captured in nickel coated (**a**) or adsorbed in conventional ELISA plates (**b**). IgG titers in Per09, Sin16, Hels823, and Ind11 N2 immune sera were determined using 3-fold serial dilutions and expressed as endpoint titers. Dots represent experimental duplicates of the pooled sera. Dashed lines represent the limit of detection.

**Supplementary figure 3**. **Pathogenicity of A/Singapore/1/1957 in DBA/2J mice**. (**a**) Relative body weight and survival of mice inoculated with a 3-fold serial dilution of parental Sin57. (**b**) Relative weight and survival of mice inoculated with a 3-fold serial dilution of mouse-adapted A/Singapore/1/1957.


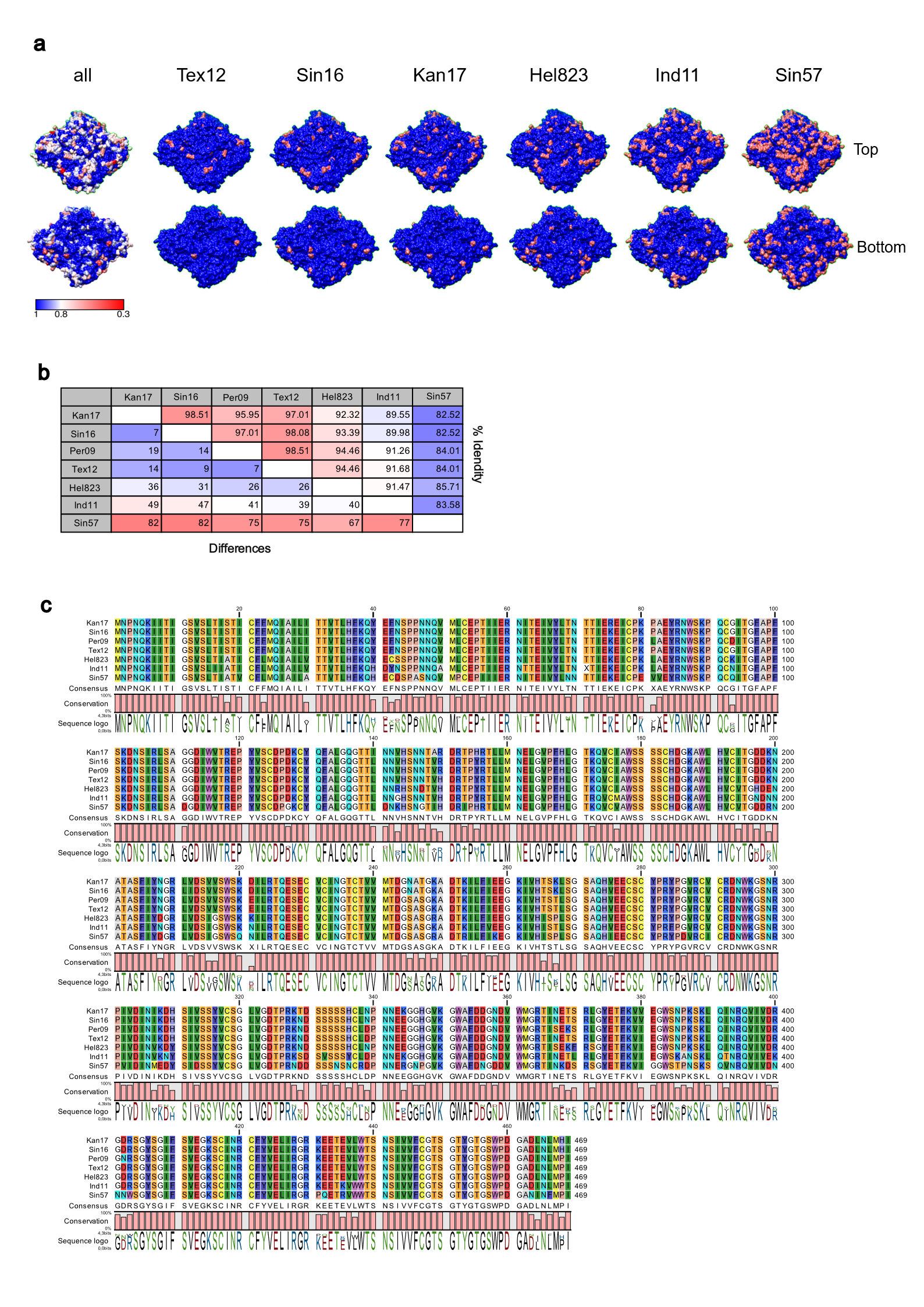


**Supplementary figure 4. Amino acid conservation among N2s used in the study.** (a) Conservancy among All N2s, Tex12, Sin16, Kan17, Hel823, Ind11 and Sin57 relative to NA Per09 shown on surface representation of A/Perth/16/2009 N2 (PBD ID: 6BR5). (b) Pairwise amino acid sequence identity matrix indicating the percentage identity and differences. (c) Amino acid sequence alignment.
